# Relationship between levels of the heavy metals lead, cadmium and mercury, and metallothionein in the gills and stomach of *Crassostrea iredalei* and *Crassostrea glomerata*

**DOI:** 10.12688/f1000research.14861.1

**Published:** 2018-08-10

**Authors:** Asus Maizar Suryanto Hertika, Kusriani Kusriani, Erlinda Indrayani, Rahmi Nurdiani, Renanda B. D. S. Putra

**Affiliations:** 1 Faculties of Fisheries and Marine Science, University of Brawijaya, Malang, Indonesia

**Keywords:** Heavy metal, Biomarker, Metallothionein, Crassostrea iredalei and Crassostrea glomerata

## Abstract

**Background:** The objective of this study was to compare the levels of heavy metals (Pb, Hg, and Cd) and metallothionein (MT) in the gills and stomach of two species of mussels ( *Crassostrea iredalei* and *Crassostrea glomerata*), and to observe the ability of the mussels to absorb the heavy metals Pb, Hg and Cd present in the water.

**Methods:** The mussels were obtained from Mayangan, Kenjeran and Gresik ports, East Java, Indonesia. MT levels were determined using ELISA. Heavy metal levels of Pb, Hg and Cd were assayed using atomic absorption spectrophotometry.

**Results:** The levels of Pb and Cd in water were below the maximum permissible levels for local water quality standards. By contrast, the level of Hg in the water was above the maximum permissible levels for water quality standards. At Mayangan Port (Station 1), the level of Pb was higher than Hg and Cd. Levels of MT and heavy metals varied greatly among of *C. iredalei* and *C. glomerata* individuals, but were always higher in the gills than in the stomach. The highest MT level (160,250 ng/g) was observed at Kenjeran Port (Station 2). MT levels were shown to be significantly associated with heavy metal level ( *P*<0.0001).

**Conclusions:** This result indicates that MT may be responsible for the sequestration of these heavy metals, as has already been observed in terrestrial animals.

## Introduction

Pollution occurring in coastal environments is mainly caused by human and industrial activity, and has become a matter of concern over the last few decades ^1, 2^. Common chemical pollutants, including heavy metals, such as Cd, Hg and Pb, are considered to be toxic and harmful pollutants. Heavy metal pollution may have devastating effects on both the ecological environment and aquatic organisms ^3^. The organisms and biomass contaminated with heavy metals could eventually affect human health ^4– 6^.

Accumulation of heavy metals in marine organisms can be considered as an important pathway of the transfer of heavy metals ^7^. As a marine bivalve, the suspension-feeding activity of mussels represent the main pathway for heavy metal uptake and accumulation ^8, 9^. Mussels are suspension feeders, both aqueous and dietary, such as material suspended from sediments consisting of high-molecular-weight substances, microorganisms, fecal pellets and detritus ^10, 11^. Mussels are commonly used to assess the eco-toxicological effects of the products released by anthropogenic activities ^12– 14^. In a previous study, mussels were used to evaluate *in situ* metal contamination in wastewater effluence and other aquatic ecosystems ^15, 16^. The concentration of metal in the tissue of mussels increased concomitantly with the elevation of metal absorption or uptake, and the various metal bioaccumulation levels were observed in different tissues of mussels ^17, 18^.

Metallothionein (MT) plays a prime role as a response to heavy metal that accumulated in mussel. MT is well-known as a biomarker of heavy metal pollution in aquatic organisms ^19– 22^. MT is a heavy-metal-binding protein mostly synthesized by bivalves as a response to the presence of heavy metals. It functions to remove divalent bonds formed by heavy metals and metalloids ^23^. In another study by Gagnon *et al.* in 2014 ^24^, MT was also found to bind reactive oxygen species such as nitric oxide, therefore released during the process of inflammation. Furthermore, the accumulation of heavy metals may induce oxidative stress which promotes the substantial impairment of lipid function in mussel tissues. Furthermore, the accumulation of heavy metals in mussels can also directly affect the health of the bivalve without elevating heavy metal concentration in bivalve tissues ^25^.

In a previous study by Raspor *et al.* ^26^, *Crassostrea iredalei* and *Crassostrea glomerata* were used as biomarkers for monitoring heavy metal pollution based on MT level. MT was synthesized differently among bivalve tissues. The gills and stomach of the bivalve were used to examine the heavy metal pollutant levels. However, the specific relationship between each heavy metal (Pb, Hg, Cd) and MT levels in the gills and stomach is largely unknown. In the present study, we therefore determined the relationship between the accumulation of heavy metals (Pb, Hg Cd) and MT levels in the gill and stomach of *Crassostrea iredalei* and *Crassostrea glomerata* obtained from coastal environments in East Java, Indonesia (Mayangan Port, Kenjeran Beach, and Gresik Port). This study can also be used to assess the management policy strategies of East Java coastal in an effort to minimize coastal environment pollution.

## Methods

### Sampling of mussels

Mussels ( *C. glomerata* and *C. iredalei*) were collected from the north coast area of East Java such as Mayangan Port (Probolinggo), Kenjeran Beach (Surabaya), and Gresik Port (Gresik). Sub-stations 1,2 and 3 in Mayangan are located geographically at 7°44’12.70’’ S, 113°12’41.54’’ E; 7°43’39.94’’ S, 113°13’19.87’’ E; and 7°44’18.08’’ S, 113°13’40.44’’ E, respectively. At Kenjeran Beach Surabaya, sub-stations 1, 2 and 3 are located geographically at 7°14’03.67’’ S, 112°47’44.28’’ E; 7°13’52.73’’ S, 112°47’38.72’’ E; and 7°13’41.38’’ S, 112°47’31.14’’ E, respectively. Sub-stations 1, 2 and 3 of Gresik Port are located geographically at 7°13’27.61’’ S, 112°40’57.90’’ E; 7°13’28.98’’ S, 112°41’10,24’’ E; 7°13’23.13’’ S, 112°40’21.07’’ E, respectively. The three samples of gill and stomach tissue of both *C. glomerata* and *C. iredalei* were collected from three sub-stations during the lowest low tide at the intertidal area of each sampling station.

### Heavy metal examination

Heavy metals (Pb, Cd, and Hg) were examined from samples of seawater and tissues of mussels (gill and stomach) from each sampling station. The seawater was collected and filtered through a 0.45-mm polycarbonate membrane Nucleopore filter (Millipore) into a glass bottle to prevent contamination or metal absorption. Nitric acid was added to the seawater to obtain a pH lower than 2. The tissue samples were prepared according to established method ^27^. In order samples can be oxidized completely and to destruct organic substances at low temperatures to avoid evaporating mineral loss, 0.2 g of gill or stomach tissues was added to 2 ml HNO _3_ (1 M) (Fluka) and incubated for 30 min. Afterward, the tissue samples were centrifuged for 15 min at 12,000 *g*. The supernatant was collected and the heavy metals content were determined using a Varian A220 Atomic Absorption Spectrophotometer (Varian, Inc.).

### MT determination

Briefly, 0.5 g gills and stomach organs of *C. iredalei* and *C. glomerata* were washed three times with PBS solution and frozen at −20°C. Frozen tissues were then crushed and mixed with 3 ml homogenization buffer (0.5 M sucrose, 20 mM Tris-HCl buffer, pH 8.6, containing 0.01% β-mercaptoethanol). The homogenate was then centrifuged at 30.000 *g* for 20 min to get supernatant containing MT. A total of 1.05 ml cold ethanol and 80 ml chloroform were then added per 1 ml of supernatant and this was centrifuged at 6000 *g* for 10 min. The pellet produced was washed using ethanol, chloroform and homogenization buffer at ratio of 87:1:12, respectively. The pellet was then dried using nitrogen gas to complete evaporation before it was re-suspended in 300 ml of 5 mM Tris-HCL, 1 mM EDTA, pH 7. The concentration of the MT fraction was reduced to 4.2 ml (0.43 mM) by addition of 5,5 dithiobis(2-nitrobenzoic acid) in 0.2 M phosphate buffer, pH 8. The sulfhydryl concentration was reduced by incubating the mixture for 30 min at room temperature.

The MT content was determined using indirect ELISA. The coating antigen to coating buffer ratio used was 1:40. The solution was incubated overnight at 4°C. Afterward, the plate was washed six times using 100 μl PBS/0.2% Tween solution. Next, 100 μl primary antibody of IgG1 rabbit anti-MT (1:400) (Santa Cruz Biotechnology, Cat# J0410) was added to assay buffer. ELISA plate was then incubated at room temperature for 2 hours before it was washed six times with 200 μl 0.2% PBS. In total, 100 μl of polyclonal secondary antibody of IgG biotin anti-rabbit (1:800) (Santa Cruz Biotechnology, Cat# L061) was added to assay buffer. The mixture was incubated at room temperature for 1 hour and washed 6 times with 0,2% PBS. Next, 100 μl streptavidin horseradish peroxidase (1:800) was added to the assay buffer in order to detect the reagent for primary antibodies conjugated to biotin. The solution was incubated at room temperature in shaker incubator and then washed 6 times with 200 μl of 0,2% PBS Tween after 1 hour, 100 μl blue 3,3',5,5'-tetramethylbenzidine, as substrate for horseradish peroxidase, was added to each well and the plate was incubated for 20–30 min in a dark room. A reaction was considered to have occurred if the color of the solution changed to blue, indicating the presence of MT. The reaction was stopped by adding 100 μl 1 M HCl. At this stage, the blue solution becomes yellow. The absorbance was measured using an ELISA reader at 450 nm wavelength. The results were then converted using a standard curve to obtain the MT value.

### Water quality examination

Physicochemical analyses were done according to Standard Methods ^28^. Dissolved oxygen concentration was determined by using Oxymeter (YSI PRO 20). Furthermore, pH-indicator strips Universal indicator (MERCK, CAT# HC000419) was measured pH *in situ* at the sampling stations. A Refractometer (RHS-10ATC, SINOTECH) was used to measure salinity. Temperature was determined by using thermometer-Hg.

### Data analysis

Data analysis was performed using SPSS version 16. The association between Pb, Cd and Hg contents with MT value was determined using multiple regressions with variable Y was density or intensity, variable X1 was Pb content, X2 was Cd content and X3 was Hg content.

## Results and discussion

### Heavy metal content in seawater

**Figure 1.**
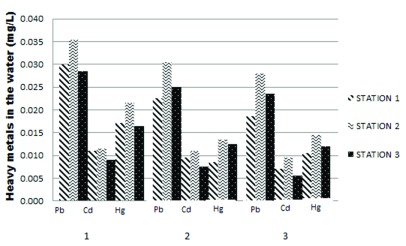
Heavy metal level (Pb, Cd, Hg) at the three stations. Station 1, Mayangan; Station 2, Kenjeran; Station 3, Gresik port.

The heavy metal content (Pb, Cd and Hg) observed at three research stations (Mayangan, Kenjeran, and Gresik port) is shown in Figure 1. The level of heavy metal Pb was higher than Hg and Cd at all three sampling stations. The highest Pb and Cd value were observed at Kenjeran at around 0.036 mg/l and 0.012 mg/l, respectively. According to the Ministerial Decree of Living Environmental No 51 Year 2004 concerning water quality standard to heavy metal content, Hg content for aquatic environments should be no more than 0.003 mg/l, Pb no more than 0.05 mg/l and Cd no more than 0.01 mg/l.

### Heavy metal analysis in gill and stomach tissue

**Figure 2.**
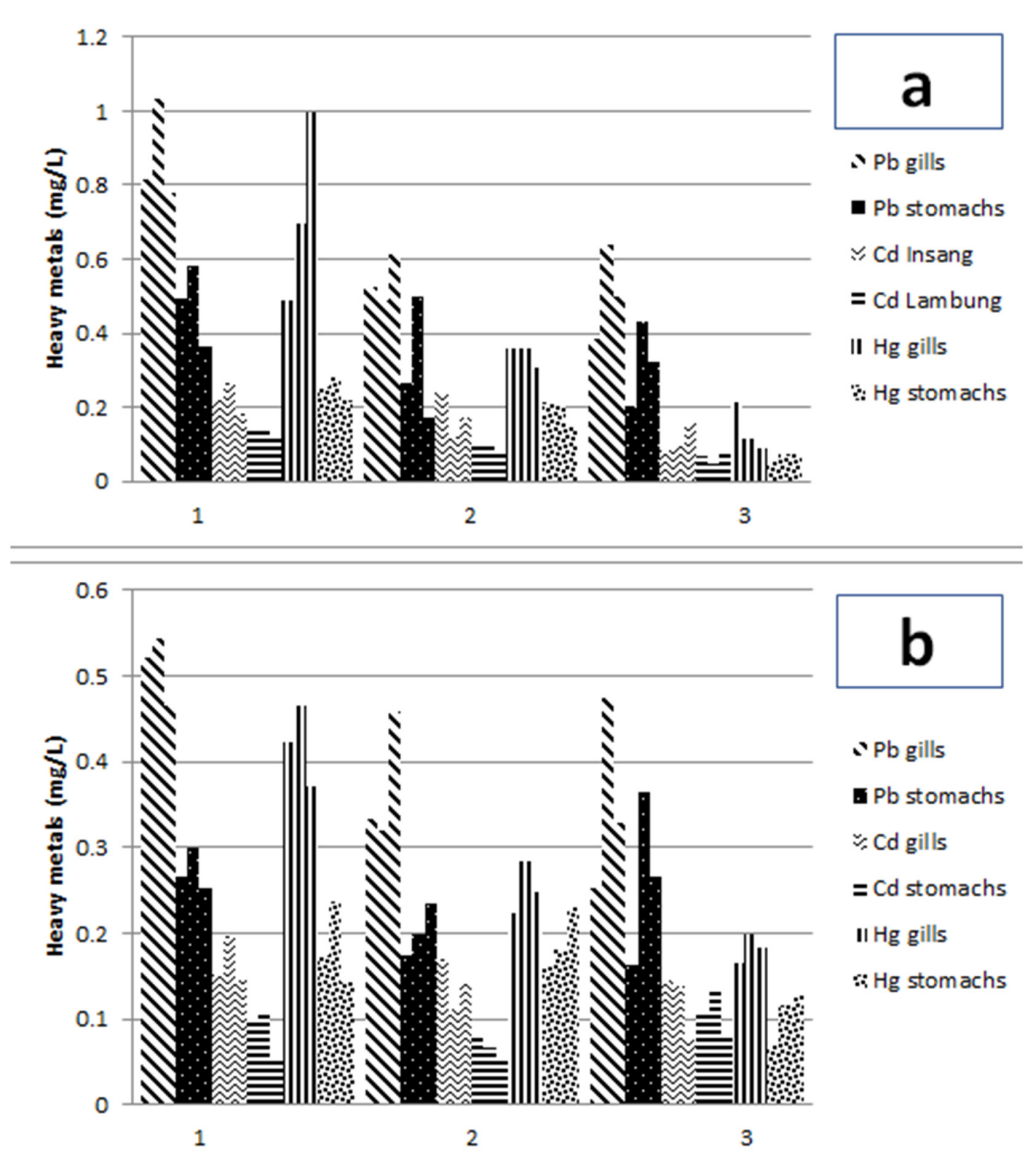
Heavy metal (Pb, Cd and Hg) content in the gills and stomach of ( **a**) *Crassostrea iredalei* and ( **b**) *Crassostrea glomerata* at the three stations. Station 1, Mayangan; Station 2, Kenjeran; Station 3, Gresik port.

The heavy metal concentration (Pb, Cd and Hg) in gill and stomach tissues of *C. glomerata and C. iredalei* is shown at Figure 2.

**Figure 3.**
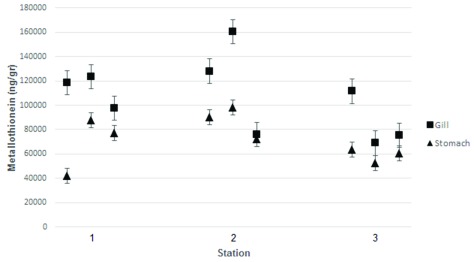
Metallothionein level (wet mass) in the gills and stomach of *Crassostrea iredalei* collected from the three stations. Station 1, Mayangan; Station 2, Kenjeran; Station 3, Gresik port.

**Figure 4.**
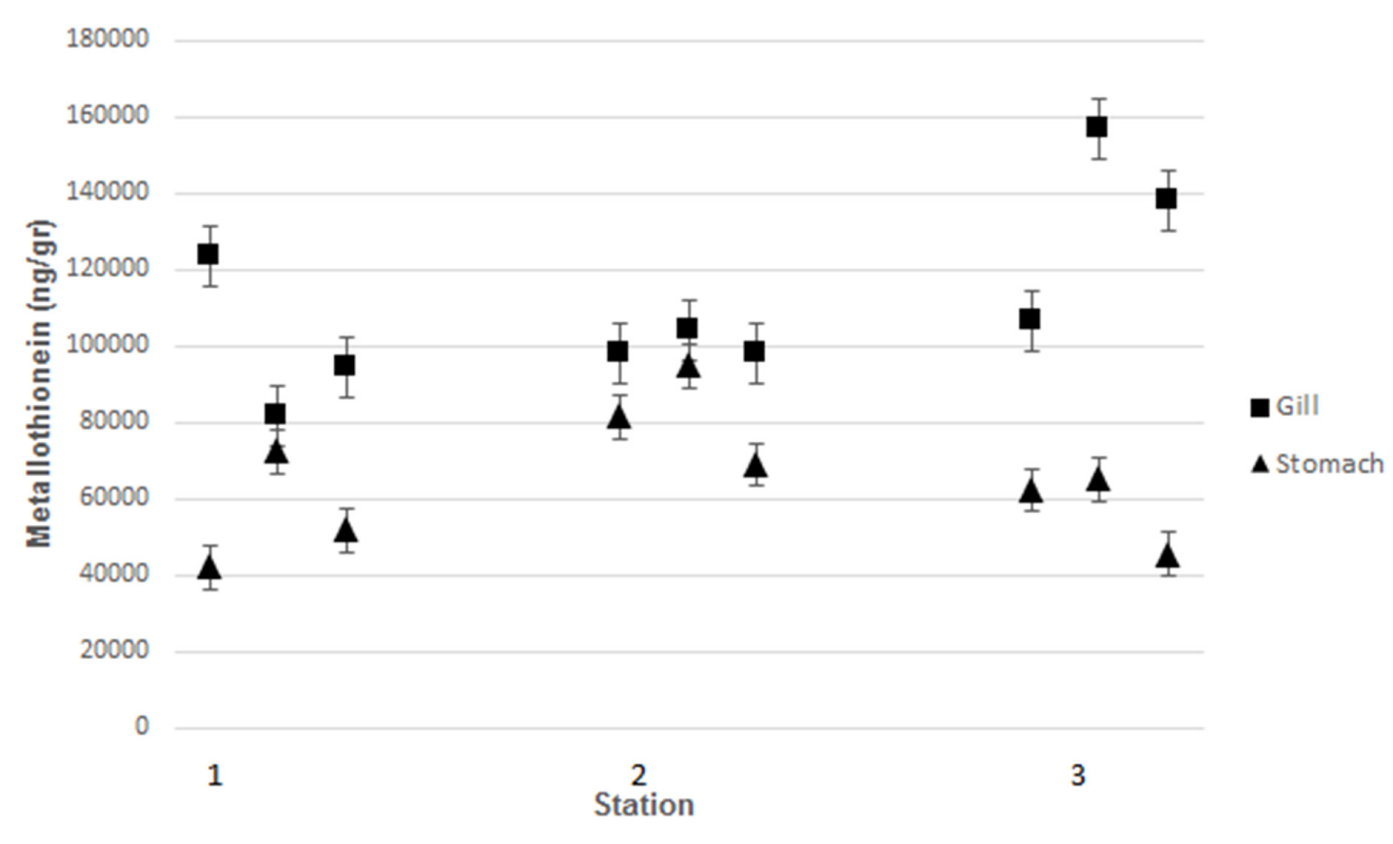
Metallothionein level (wet mass) in the gills and stomach of *Crassostrea glomereta* collected from the three stations. Station 1, Mayangan; Station 2, Kenjeran; Station 3, Gresik port.

Mussels were used as candidate to determine the heavy metal concentration in seawater because mussels are filter feeders and settled/stationary ^29^. Many studies have been conducted on the determination of the heavy metal level in mussel tissue as a pollutants monitoring tool ^30– 33^. Figure 2 shows that heavy metal levels were higher in the gills than in stomach of the mussels. The highest value of heavy metal in gill tissue of *C. iredalei* was obtained from Mayangan, with a Pb concentration 0.715–1.061 mg/l, followed by Cd at 0.168–0.269 mg/l, and Hg at 0.420–0.731 mg/l. In the stomach, heavy metal Pb was ranged at 0.352–0.600 mg/l, Cd at 0.099–0.149 mg/l, and Hg at 0.171–0.337 mg/l. Similar results were obtained from *C. glomerata* tissue. The highest value of heavy metals in gills was obtained at station 1 with Pb content 0.419–0.649 mg/l, followed by Cd at around 0.101–0.234 mg/l, and Hg 0.300–0.582 mg/l. The heavy metal levels of Pb, Cd and Hg in the stomach were 0.231–0.326 mg/l, 0.034–0.134 mg/l, and 0.077–0.308 mg/l, respectively.

### MT levels in the gills and stomach of *C. glomerata* and *C. iredalei*

Measurement of MT levels was performed using ELISA. *C. iredalei* and *C. glomereta* produced higher MT levels in the gills than in the stomach tissues The highest MT levels, around 160,250 ng/g, were observed from samples obtained from station 2 (Kenjeran). The highest MT level measured in Mayangan was 123.500 ng/g, while at Gresik port it was 111.500 ng/g.

Similar results were observed from *C. glomerata* samples. The highest MT level was obtained in gill of *C. glomereta* collected from Kenjeran at 159,000 ng/g. At Mayangan, the highest MT in the gills was around 121,800 ng/g, while at Gresik port was around of 108,900 ng/g. According to Ringwood *et al*. ^34^, there was a positive association between the level of MT and that of heavy metal pollutants. Heavy metal pollutants cause systemic damage in organisms and induce MT production ^35^. According to Rumahlatu *et al.* ^36^, MT in mussels binds heavy metals, meaning that MT can be used as an indicator of pollution. Organic materials and heavy metals in seawater can accumulate in bivalves in the gills, kidneys, and stomach. Furthermore, organic materials accumulated in the mussels are secreted through the kidney, while the heavy metals may induce synthesis of MT in gills and stomach ^37^. According to Suryono ^38^, bivalves are able to detoxify heavy metals by synthesizing MT. As heavy metal accumulate in the body of the bivalve, MT synthesis reaches its maximum level. This event can be used to monitor environmental contamination by heavy metals ^39^. Cu, Cd, and Zn in seawater have been reported to promote MT synthesis in different tissues, such as the digestive gland and gills of mussels ^40^.

### Relationship between heavy metal content of (Pb, Cd and Hg) with MT content (quantitative) in gill and stomach of *C. iredalei*

The relationship between the content of heavy metals and MT level was significant ( *P*<0.0001). According to Sungkawa ^41^, regression analysis basicly using two variables such as independent variable noted as X and dependent variable noted as Y. According to Amiard *et al.* ^20^, regression analysis can be used to determine the most important parameters affecting MT level among natural factors (salinity, sex, season, total concentration protein) or contaminant factors. In the present study, multiple regression analysis of heavy metal concentration in seawater and the level of MT in the gills of *C. iredalei* resulted the equation as: Y = 52,051.866 – 30,919.060 ( *X _1_*) + 139,589.243 ( *X _2_*) + 146,797.196 ( *X _3_*). The results showed that an increase in Pb ( *X _1_*) by 1 ppm decreased MT level by 30,919.060 ng/g. Furthermore, an increase of Cd ( *X _2_*) by 1 ppm would increase MT level to around 139,589.243 ng/g. Moreover, an increase in the level of Hg ( *X _3_*) by 1 ppm would increase MT level by 146,797.196 ng/g.

In addition, we investigated the relationship between the level of heavy metals in seawater and MT levels in the stomach of *C. iredalei* was significantly associated ( *P*<0.0001). The following multiple regression equation was produced: Y = 23,320.8 – 53,844.1 ( *X _1_*) +268,073 ( *X _2_*) + 658,306 ( *X _3_*). The results showed the increased of Pb ( *X _1_*) by 1 ppm would reduce the MT level to 53,844.1 ng/g. Furthermore, an increase of Cd ( *X _2_*) and Hg ( *X _3_*) concentration by 1 ppm would elevate the MT level to around 268,073 ng/g and 658,306 ng/g, respectively.

Determining pollution levels using MT has become of great interest in the marine environment, and MT is seen as potential biomarkers of metal exposure in molluscs and other marine organisms ^42^. In previous study, MT were found and quantified in various tissues of *Mytilus galloprovincialis*, especially in the digestive gland and gills ^43^. The results of a prior study showed that the MT content in the digestive gland of *Mytilus galloprovincialis* was significantly higher than that in the gills ^44^.

### Relationship between heavy metal content (Pb, Cd and Hg) towards MT content (quantitative) in gill and stomach at *C. glomerata*

We observed the relationship of heavy metal level with MT level in gill and stomach of *C. glomerata*. The heavy metal level has significant association ( *P*<0.0001) with MT level in gill. Using multiple regression analysis, we obtained the following equation: Y = 48,092.338 – 29,404.578 ( *X _1_*) +223,621.464 ( *X _2_*) + 144,733.404 ( *X _3_*). The results showed that an increase in Pb ( *X _1_*) concentration by 1 ppm decreased the MT level in gills to 29,404.578 ng/g. An increased in the Cd ( *X _2_*) concentration of 1 ppm elevate of MT level to 223,621.464 ng/g and the increased of Hg ( *X _3_*) concentration 1 ppm elevated MT level to 144733.404 ng/g.

Furthermore, the heavy metal level has significant association ( *p-value*, 0.0001< 0.05) with MT level in stomach. On the basis of the results of multiple regression of heavy metal content in stomach of *C. glomerata* the following equation was obtained: Y = 15,279.782–4,991.670 ( *X _1_*) +105,058.703 ( *X _2_*) + 225,262.150 ( *X _3_*). The results showed the increased of Pb ( *X _1_*) concentration by 1 ppm would decrease MT level to 4,991.670 ng/g. Increasing Cd ( *X _2_*) and Hg ( *X _3_*) concentration by 1 ppm would elevate MT level to 105,058.703 ng/g and 225,262.150 ng/g, respectively.

The presence of heavy metals affected the level of MT because it has function to detoxify heavy metals. According to Rumahlatu *et al.* ^35^, MT functions as a metal-binding protein that accumulates in the mussel body and can be used as a marker of heavy metal pollutants. Although many aquatic organisms produce MT, making them candidates for modeling heavy metal pollution, mussels have been shown to accumulate higher levels of heavy metals than other species because they are filter feeders. Thus, mussels are good candidates for investigation the heavy metal pollutant levels through levels of MT ^45^. The differences in tissue distribution may be due to the changes in metabolism of protein or to protein levels in the digestive gland of mussels ^46^. MT concentrations increased in the clam *Ruditapes philippinarum* and green mussel *Perna viridis* tissues after they were exposed to increasing concentrations of Cd in the laboratory ^47^.

### Water quality parameters

The water quality of seawater (temperature, acidity level (pH), dissolved oxygen (DO) and salinity at each station is shown in Table 1.

**Table 1.** Water quality in each station.

Water quality parameter	Sub station	Mayangan	Kenjeran	Gresik Port
Temperature, °C	1	29	29	23.4
	2	31	31	23.3
	3	30	31	30
pH	1	9	9	9
	2	9	9	9
	3	9	9	9
Dissolved O _2_, mg/l	1	5.38	3.38	8.9
	2	4.19	5.2	8
	3	8.17	5.1	5
Salinity, ppt	1	32	32	29
	2	33	17	21
	3	33	23	16

ppt, parts per thousand

The present study showed that the temperature of seawater ranged between 23.4–31°C. MT accumulation in the mussel body increases significantly during the dry season ^48^. Temperature has a notable influence on heavy metal solubility. Increasing water temperature leads to the increased solubility of heavy metal solubility, which is toxic ^49^. According to the Water Quality Standard of Ministerial Decree of Living Environment No.51 year 2004, normal temperature for the marine biota environment ranges between 28 and 30°C. In the present study, the pH value obtained was around 9. The pH was not suitable for bivalves because while the waters pH is high, the heavy metal in seawaters will be settled at the bottom and will absorbed by bivalves ^50^, leading to death of the bivalve. The salinity result obtained ranged between 17 and 33 parts per thousand (ppt). According to KMNLH No. 51 Year 2004, the standard quality of seawater salinity is around 27–33 ppt. Distribution and concentration of heavy metal in waters environment will increase along with salinity value increase ^51^. The dissolved oxygen concentration observed in the present study ranged from 3.85 to 8.9 mg/l. The dissolved oxygen also influences to heavy metal toxicity, as lower dissolved oxygen cocnentration promotes the elevation of toxicity of heavy metals in the water ^52^.

Raw data for heavy metal levels contained in mussels taken from each location Data are organized by the Figure in which they appear. Click here for additional data file. Copyright: © 2018 Hertika AMS et al. 2018 Data associated with the article are available under the terms of the Creative Commons Zero "No rights reserved" data waiver (CC0 1.0 Public domain dedication).

## Conclusion

On the basis of the results of this study, we conclude that there is significant relationship between heavy metal concentration in the seawater and MT levels in the gills and stomach of *C. glomerata* and *C. iredalei* ( *p-value*, 0.0001< 0.05).

## Data availability

The data referenced by this article are under copyright with the following copyright statement: Copyright: © 2018 Hertika AMS et al.

Data associated with the article are available under the terms of the Creative Commons Zero "No rights reserved" data waiver (CC0 1.0 Public domain dedication). 

Dataset 1. Raw data for heavy metal levels contained in mussels taken from each location. Data are organized by the Figure in which they appear. DOI: http://doi.org/10.5256/f1000research.14861.d213155 ^53^
