# Supplementary material for: Relationship between levels of the heavy metals lead, cadmium and mercury, and metallothionein in the gills and stomach of Crassostrea iredalei and Crassostrea glomerata
Source: F1000Res. 2018 Aug 10;7:1239. [Version 1] doi: 10.12688/f1000research.14861.1 (PMC6124388; doi:10.12688/f1000research.14861.1)
Supplement: Peer review history

# Peer review: Referee response for version 1

**DOI:** 10.5256/f1000research.16176.r37088

**Published:** 2018-08-31

**Authors:** Akhmad Taufiq Mukti

**Affiliations:** 1 Department of Fish Health Management and Aquaculture, Faculty of Fisheries and Marine, Universitas Airlangga (UNAIR), Surabaya, Indonesia

Approved with notes:

1. In the ‘Introduction, the final sentence of paragraph 1 is not related to the previous sentence. I suggest that the authors need a statement about the direct and indirect influences of heavy metals on ecological environment and aquatic organisms.
2. In ‘Methods’, I suggest that the authors describe the reasons for selecting a sampling location.
3. In ‘Methods’, I suggest that the authors describe the reasons for selecting gills and stomach as a sample organs.
4. In ‘Results and discussion’, the authors have not described a discussion based on results in “Heavy metal content in seawater” and in “Heavy metal analysis in gill and stomach.
5. The authors used heavy metals of Pb, Hg and Cd, why use these three heavy metals as indicators, not other heavy metals, maybe the authors could be explain the reason?

I have read this submission. I believe that I have an appropriate level of expertise to confirm that it is of an acceptable scientific standard.

# Peer review: Referee response for version 1

**DOI:** 10.5256/f1000research.16176.r37089

**Published:** 2018-08-20

**Authors:** Ima Yudha Perwira

**Affiliations:** 1 Water Resource Management, Faculty of Marine and Fisheries, Udayana University, Jimbaran, Indonesia

**Summary**

 This study is studying about comparison of heavy metal level (Pb, Hg, and Cd) in the gills and stomach of two different mussel species: *Crassostrea iredalei* and *Crassostrea glomerata*. This study also explains about the absorbance capacity of those mussels to Pb, Hg, and Cd. The result showed that MT level in the gills in both mussels are higher than that in the stomach, which is correspond to the higher heavy metal content in the gill than that in the stomach. This result indicate the relation between the MT production and heavy metal level in mussel.

 Question 1: This study is clear and accurate. The literature used by the author are also correspond to the article.

 Question 2: This study showed appropriate design. The selection of study site in several place (Probolinggo, Surabaya, and Gresik) is suitable, since the high population of heavy metal industries in those area.

 Question 3:  The methods and analysis used by the author is proper to be use by another author. The using of ELISA technique is common to be used to analyze MT level in marine bivalves.

 Question 4: The statistical analysis and its interpretation are also correct. Therefore, there is no doubt in it.

 Question 5: The source data underlying the results available to ensure full reproducibility.

 Question 6: The author have concluded the results in very simple and easy to be understand sentence.

I have read this submission. I believe that I have an appropriate level of expertise to confirm that it is of an acceptable scientific standard.
